# Crosslinked poly(methyl methacrylate) with perfluorocyclobutyl aryl ether moiety as crosslinking unit: thermally stable polymer with high glass transition temperature

**DOI:** 10.1039/c9ra10166g

**Published:** 2020-01-09

**Authors:** Yang Li, Hao Guo

**Affiliations:** Department of Chemistry, Fudan University 2005 Songhu Road Shanghai 200438 People's Republic of China hao_guo@fudan.edu.cn

## Abstract

Crosslinked poly(methyl methacrylate) (PMMA) with high glass transition temperature (*T*_g_) and thermal decomposition temperature was prepared by simple thermal crosslinking of PMMA-containing random copolymers bearing aryl trifluorovinyl ether (TFVE) moieties. A methacrylate monomer consisting of aryl TFVE moiety, 4-((1,2,2-trifluorovinyl)oxy)phenyl methacrylate (TFVOPMA), was first synthesized followed by radical copolymerization with methyl methacrylate (MMA) initiated by AIBN, providing the random copolymer containing aryl TFVE moieties, poly(4-((1,2,2-trifluorovinyl)oxy)phenyl methacrylate)-*co*-poly(methyl methacrylate) (PTFVOPMA-*co*-PMMA). Finally, crosslinked PMMA polymer with perfluorocyclobutyl (PFCB) aryl ether moieties as crosslinking units was obtained by [2π + 2π] cycloaddition reaction of aryl TFVE moieties in PTFVOPMA-*co*-PMMA copolymer. Thermal properties of both PTFVOPMA-*co*-PMMA and crosslinked PTFVOPMA-*co*-PMMA were examined by TGA and DSC. Compared to pure PMMA, *T*_g_ of PTFVOPMA-*co*-PMMA increased by 15.1 °C and no *T*_g_ was found in the DCS test of the crosslinked PTFVOPMA-*co*-PMMA. Thermal decomposition temperature (*T*_d,5%_) of crosslinked PMMA was 47 °C higher than that of pure PMMA. Furthermore, the water absorption of crosslinked PMMA film greatly reduced in comparison with that of pure PMMA.

## Abstract 2

PMMA-based copolymer containing aryl TFVE moieties prepared by copolymerization of MMA and TFVOPMA monomer containing aryl TFVE group and thermal cross-linked by [2π + 2π] cycloaddition reaction of aryl TFVE moieties.

## Graphical abstract

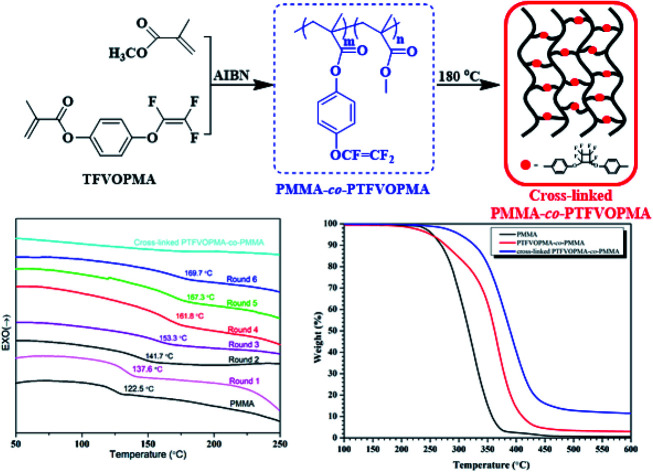

## Introduction

Poly(methyl methacrylate) (PMMA) is a kind of transparent polymeric material possessing diverse excellent properties such as superior light transmittance, light weight, chemical stability, weathering corrosion resistance, electrical insulation and good processability, which make PMMA widely applied in many fields including aerospace, building construction and optical instruments.^1–4^ However, the glass transition temperature (*T*_g_, 100 °C) and heat-deformation temperature of PMMA are relatively low so as to limit its applications.^5,6^ In order to improve *T*_g_ and the thermal stability of PMMA, investigators have explored various methods,^3,7–15^ including the copolymerization of methyl methacrylate (MMA) with comonomers bearing rigid or bulky groups to overcome the miscibility puzzle, or the formation of a three-dimensional network structure by the addition of crosslinking agent. Wilkie *et al.* prepared MMA–DVB crosslinked polymers using divinylbenzene (DVB) as crosslinking agents and the degradation temperature (*T*_d,5%_) of resulting polymers increased from 162 °C to 294 °C.^12^ Kuo *et al.* suggested an approach to raise the *T*_g_ of PMMA through copolymerization with methacrylamide (MAAM) since hydrogen-bonding interactions exist between these two monomer segments.^16–19^

Fluoropolymers have attracted much curiosity in material science due to their unique properties including thermal stability, chemical resistance, flame retardancy, superior electrical insulating ability, low dielectric constant and refractive index, and unique surface property.^20–22^ To avoid low processability of perfluoropolymers, partially-fluorinated polymers like poly(vinylidene fluoride) (PVDF),^4,5^ alternating ethylene-tetrafluoroethylene (ETFE)^23^ and alternating ethylene-chlorotrifluoroethylene (ECTFE) copolymers,^24,25^ have been prepared and exhibited improved processability. Among partially-fluorinated polymers, perfluorocyclobutyl (PFCB) aryl ether polymers^26–31^ are an intriguing class of fluoropolymers, which not only retain the general outstanding properties of fluoropolymers originating from the low polarity, strong electronegativity and small van der Waals radius of fluorine atom and strong C–F bond, but also possess many other advantages such as improved processability and optical transparency, structural versatility and tunable properties. These PFCB aryl ether-based polymers have been studied on the potential application in photonics, polymer light-emitting diodes, proton exchange membrane in fuel cell and atomic oxide resistance materials on spacecraft *etc.*^32–40^

Inspired by the fact that PFCB aryl ether-based polymers possess high heat resistance and optical transparency, Prof. Huang of Shanghai Institute of Organic Chemistry *et al.* prepared a series of PFCB-containing copolymers with high heat resistance.^41–43^ The resulting polymethacrylate bearing sulfonyl functionality exhibits excellent thermal stability (*T*_d_ > 300 °C) and polyimide (PFCBBPPI) containing PFCB biphenyl ether moieties shows excellent thermal stability and high transparency. PFCB aryl ether polymers are most commonly prepared through a thermal [2π + 2π] cycloaddition of aryl trifluorovinyl ethers (TFVE) at temperatures of between 150 °C and 200 °C without additional agent and no condensation byproducts are produced. Thus, TFVE group may act as a crosslinkable group and the crosslinked region, perfluorocyclobutyl (PFCB) moieties, may give rise to better chemical resistance. Lee *et al.* synthesized a novel fluorinated aromatic polyether monomer containing TFVE group and the resulting crosslinked polymers after thermal crosslinking showed higher *T*_g_ (239–271 °C).^44^

**Scheme 1:**
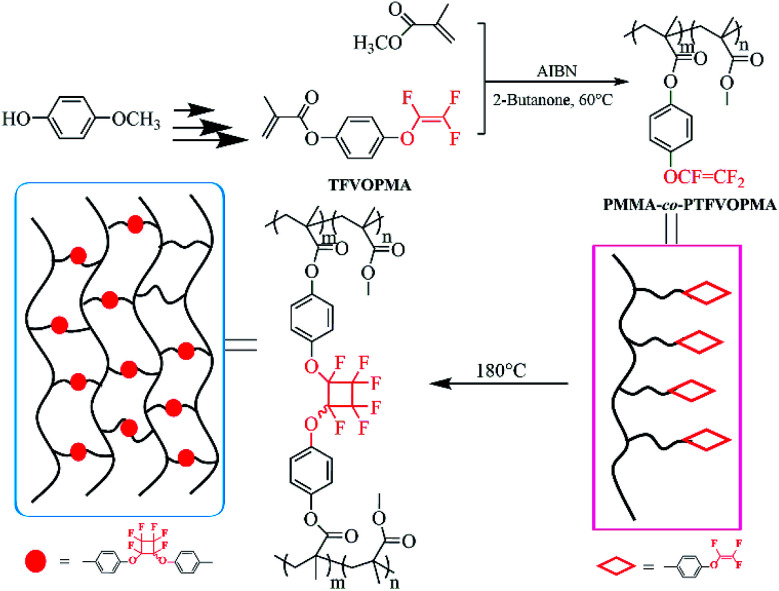
Schematic illustration of preparation of transparent crosslinked poly(methyl methacrylate) copolymers with PFCB aryl ether as crosslinking unit.

All aforementioned discussions intrigued us to synthesize methacrylate monomer containing TFVE group, which can be copolymerized with MMA to provide polymethacrylate copolymers containing TFVE groups so that the subsequent thermal cycloaddition of TFVE groups can produce crosslinked polymethacrylate copolymers with PFCB aryl ether moieties as the crosslinked region. The resulting crosslinked polymethacrylate copolymers may have higher thermal stability and good optical transparency because of the below points: (1) PFCB aryl ether-based polymers exhibit higher heat resistance and good transparency, (2) PFCB aryl ether moieties with large volume may decrease the activity of PMMA main chain, which might increase the heat resistance of resultant polymers, (3) three-dimensional network structure may limit the mobility and rotation ability of polymeric chain, which benefits heat resistance of resultant polymers. Herein, we report the preparation of transparent crosslinked poly(methyl methacrylate) (PMMA) copolymers with high *T*_g_ and high *T*_d_*via* copolymerization of MMA with a methacrylate monomer containing TFVE moiety, 4-((1,2,2-trifluorovinyl)oxy)phenyl methacrylate (TFVOPMA), followed by thermal crosslinking process as shown in Scheme 1. These resultant PMMA-based copolymers containing TFVE or PFCB aryl ether groups were investigated in detail by NMR, FT-IR, differential scanning calorimetry (DSC) and thermogravimetry analysis (TGA).

## Experimental

### Materials

Dimethylsulfoxide (DMSO, Alfa Aesar, 99%) was dried over CaH_2_ and distilled under reduced pressure prior to use. Triethylamine (TEA, Alfa Aesar, 99%) was dried over KOH and distilled over CaH_2_ under N_2_ prior to use. Granular zinc was activated by washing in 0.1 N HCl followed by drying at 140 °C *in vacuo* for 10 h. 2,2′-Azobis(isobutyronitrile) (AIBN, Aldrich, 98%) was recrystallized from anhydrous ethanol. Dichloromethane (DCM) and acetonitrile was dried over CaH_2_ and distilled under N_2_ prior to use. 2-Butanone was refluxed with KMnO_4_ and distilled under CaCl_2_ and KMnO_4_ prior to use. Methyl methacrylate (MMA, Aldrich, 99%) was washed with 5% aqueous NaOH solution to remove the inhibitor, then washed with water, dried over CaCl_2_ and distilled twice *in vacuo* from CaH_2_ prior to use. 1,2-Dibromotetrafluoroethane was prepared by condensing equimolar amounts of Br_2_ and tetrafluoroethylene at −195 °C followed by warming up to 22 °C according to a previous report.^1^ 4-methoxylphenol (Aldrich, 99%), caesium carbonate (Aldrich, 99%), boron tribromide (BBr_3_, Alfa Aesar, 99%) and methacryloyl chloride (Alfa Aesar, 97%) were used as received unless otherwise specified.

### Measurements

^1^H, ^13^C and ^19^F NMR spectra of intermediates were recorded on a JEOL resonance ECZ 400S spectrometer (400 MHz) in CDCl_3_. Tetramethylsilane (TMS) and CDCl_3_ were used as internal standards for ^1^H and ^13^C NMR, respectively; CF_3_CO_2_H was used as an external standard for ^19^F NMR. FT-IR spectra were recorded on a Nicolet AVATAR-360 FT-IR spectrophotometer with a resolution of 4 cm^−1^. Number-average molecular weights (*M*_n_) and molecular weight distributions (*M*_w_/*M*_n_) were obtained on a conventional gel permeation chromatography (GPC) system equipped with a Waters 515 Isocratic HPLC pump, a Waters 2414 refractive index detector, and a set of Waters Styragel columns (HR3 (500–30 000), HR4 (5000–600 000) and HR5 (50 000–4 000 000), 7.8 × 300 mm, particle size: 5 μm). GPC measurement was carried out at 35 °C using tetrahydrofuran (THF) as eluent with a flow rate of 1.0 mL min^−1^. The system was calibrated with linear polystyrene standards. Differential scanning calorimetry (DSC) was performed on a TA Q200 DSC instrument in N_2_ with a heating rate of 10 °C min^−1^. Thermogravimetry analysis (TGA) was conducted on a TA Discovery TGA 55 thermal analysis system in N_2_ with a heating rate of 10 °C min^−1^. UV/vis spectra were acquired on a Hitachi U-2910 spectrophotometer.

### Synthesis of 4-((1,2,2-trifluorovinyl)oxy)phenyl methacrylate

**Scheme 2:**
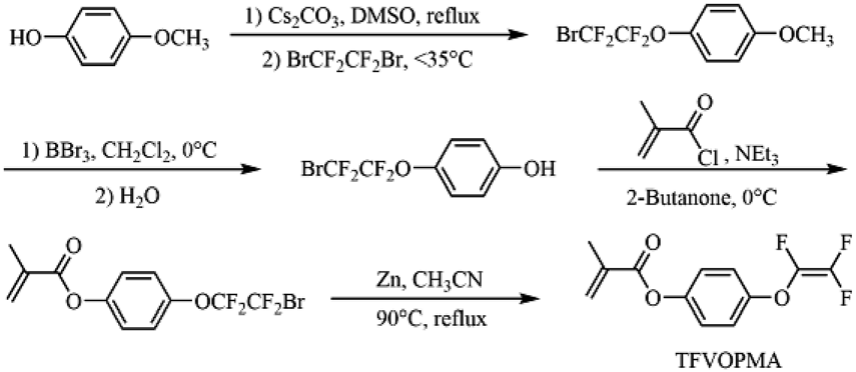
Schematic illustration of synthesis of TFVOPMA monomer.

4-((1,2,2-Trifluorovinyl)oxy)phenyl methacrylate (TFVOPMA) was synthesized *via* four steps starting from 4-methoxylphenol (Scheme 2). 1-Methoxy-4-(2-bromo-1,1,2,2-tetrafluoroethyl)benzene was firstly prepared by fluoroalkylation reaction of 4-methoxyphenol. To a 500 mL dried three-neck round-bottom flask fitted with a condenser and a constant pressure dropping funnel, 4-methoxyphenol (20.00 g, 0.16 mol) and caesium carbonate (33.60 g, 0.19 mol) were added followed by evacuating and backfilling with N_2_ three times. Next, DMSO (200 mL) was added *via* a gastight syringe and the mixture was stirred for 30 min. 1,2-Dibromotetrafluoroethane (22.60 mL, 0.19 mol) was then dropped slowly, keeping the temperature below 35 °C. The resulting mixture was heated at 50 °C for 5 h. Finally, 500 mL of deionized water was added and the organic layer was separated and extracted by dichloromethane, washed by water and brine and then dried over magnesium sulfate (MgSO_4_). 1-Methoxy-4-(2-bromo-1,1,2,2-tetrafluoroethyl)benzene (40.69 g, 84%) was obtained by silica column chromatography using hexane as eluent. ^1^H NMR (400 MHz, CDCl_3_): *δ* (ppm): 7.23 (d, 2H, *J* = 7.24 Hz, C_6_H_2_*H*_2_), 7.03 (d, 2H, *J* = 7.02 Hz, C_6_H_2_*H*_2_), 3.82 (s, 3H, *J* = 3.84 Hz, OC*H*_3_). ^19^F NMR (376 MHz, CDCl_3_): *δ* (ppm): −68.2 (s, 2F), −86.6 (s, 2F). ^13^C NMR (101 MHz, CDCl_3_): *δ* (ppm): 55.7, 114.6, 122.8, 142.0, 155.3. EI-MS (M^+^): 123 (100), 287 (1), 289 (1).

In a pre-dried flask, 1-methoxy-4-(2-bromo-1,1,2,2-tetrafluoroethyl)benzene (3.88 g, 13 mmol) was dissolved in 300 mL of anhydrous dichloromethane. The solution was cooled to 0 °C followed by adding dichloromethane solution of BBr_3_ (25 mL, 25 mmol) dropwise within 30 min. The mixture was slowly warmed up to room temperature and stirred at room temperature for another 12 h. The resulting mixture was filtrated and the residual salt was removed by washing with dichloromethane and filtrating three times. The solvents were removed by rotary evaporation, and the residue was subjected to column chromatography (eluent: hexane) to afford 4-(2-bromo-1,1,2,2-tetrafluoroethoxy)phenol as a colorless liquid (3.15 g, 84% yield). ^1^H NMR (400 MHz, CDCl_3_): *δ* (ppm): 7.10 (d, 2H, *J* = 7.11 Hz, C_6_H_2_*H*_2_), 6.86 (d, 2H, *J* = 7.11 Hz, C_6_H_2_*H*_2_), 5.73 (s, 1H, *J* = 3.84 Hz, O*H*). ^19^F NMR (376 MHz, CDCl_3_): *δ* (ppm): −68.2 (s, 2F), −86.27 (s, 2F). ^13^C NMR (101 MHz, CDCl_3_): *δ* (ppm): 116.3, 122.9, 142.0, 154.4. EI-MS (M^+^): 109 (100), 356 (2), 358 (2).

In a pre-dried flask, 4-(2-bromo-1,1,2,2-tetrafluoroethoxy)phenol (3.70 g, 13 mmol) and triethylamine (1.49 mL, 0.015 mol) were dissolved in 300 mL of 2-butanone. The solution was cooled to 0 °C followed by adding the solution of methacryl chloride (1.48 mL, 15 mmol) in 10 mL of 2-butanone dropwise. The mixture was warmed up to room temperature and stirred for another 1 h. The salt was removed by filtration and the filtrate was washed with water and dried over MgSO_4_. The organic layer was filtrated and evaporated under reduced pressure, and then the residue was purified by flash column chromatography (eluent: hexane) on silica gel to give 3.79 g (82%) of 4-(2-bromo-1,1,2,2-tetrafluoroethoxy)phenyl methacrylate as a colorless oil. ^1^H NMR (400 MHz, CDCl_3_): *δ* (ppm): 7.12 (d, 2H, *J* = 7.13 Hz, C_6_H_2_*H*_2_), 7.06 (d, 2H, *J* = 7.03 Hz, C_6_H_2_*H*_2_), 6.24 (s, 1H, *J* = 6.24 Hz, C=CH*H*), 5.66 (s, 1H, *J* = 5.66 Hz, C=CH*H*), 1.94 (s, 3H, *J* = 2.05 Hz, C*H*_3_). ^19^F NMR (376 MHz, CDCl_3_): *δ* (ppm): −70.02 (s, 2F), −88.01 (s, 2F). ^13^C NMR (101 MHz, CDCl_3_): *δ* (ppm): 18.4, 122.7, 123.1, 127.7, 135.6, 145.9, 149.3, 165.6.

Newly activated zinc (1.98 g, 0.035 mol) was added to a dried three-neck round-bottom flask followed by evacuating and backfilling with N_2_ three times. Next, dry acetonitrile (40 mL) was added and the oil temperature was lifted to 90 °C. 4-(2-Bromo-1,1,2,2-tetrafluoroethoxy)phenyl methacrylate (15.86 g, 29 mmol) was dropped slowly into the above mixture, keeping the mixture boiling slightly. The mixture was refluxed for 10 h and after cooled to room temperature, the mixture was filtered. The salt was washed by dichloromethane to extract the product. The organic phase was collected and dried over MgSO_4_. After CH_2_Cl_2_ was evaporated, the crude product was purified by flash column chromatography (eluent: hexane) to afford 4-((1,2,2-trifluorovinyl)oxy)phenyl methacrylate (TFVOPMA) as a colorless oil (3.98 g, 53%). ^1^H NMR (400 MHz, CDCl_3_): *δ* (ppm): 7.23 (dd, 2H, *J* = 7.25 Hz, C_6_H_2_*H*_2_), 7.17 (dd, 2H, *J* = 7.17 Hz, C_6_H_2_*H*_2_), 6.31 (s, 1H, *J* = 6.35 Hz, C=CH*H*), 5.75 (s, 1H, *J* = 5.78 Hz, C=CH*H*), 2.03 (s, 3H, *J* = 2.05 Hz, C*H*_3_). ^13^C NMR (101 MHz, CDCl_3_): *δ* (ppm): 18.1, 116.7, 123.2, 127.2, 135.6, 147.7, 165.6. ^19^F NMR (376 MHz, CDCl_3_): *δ* (ppm): −120.1 (dd, 1F), −126.9 (dd, 1F), −134.49 (dd, 1F). FT-IR (KBr): *ν* (cm^−1^): 2962, 2936, 2856, 1832, 1740, 1640, 1501, 1318, 1276, 1181, 1140, 947, 876, 812, 752.

### Copolymerization of TFVOPMA and MMA

AIBN (17.8 mg, 0.108 mmol) was firstly added to a 25 mL Schlenk flask (flame-dried under vacuum prior to use) sealed with a rubber septum for degassing. Next, TFVOPMA (1.28 g, 5 mmol), MMA (0.50 g, 5 mmol) and 2-butanone (3.4 mL) were introduced *via* a gastight syringe. The solution was degassed by three cycles of freezing–pumping–thawing followed by immersing the flask into an oil bath preset at 60 °C to start the polymerization. The polymerization was terminated by putting the flask into liquid nitrogen after 3 h. After repeated purification by dissolving in 2-butanone and precipitating in ethanol three times, 1.39 g (78%) of white powder, poly(4-((1,2,2-trifluorovinyl)oxy)phenyl methacrylate)-*co*-poly(methyl methacrylate) (PTFVOPMA-*co*-PMMA), was obtained after drying *in vacuo* at 30 °C. GPC: *M*_n_ = 50 900 g mol^−1^, *M*_w_/*M*_n_ = 1.84. ^1^H NMR (400 MHz, CDCl_3_): *δ* (ppm): 0.84, 1.02, 1.18 (3H, C*H*_3_), 1.85–2.04 (4H, C*H*_2_), 3.58 (3H, OC*H*_3_), 7.11 (4H, C_4_H_2_*H*_2_). ^19^F NMR (376 MHz, CDCl_3_): *δ* (ppm): −119.2 (m, 1F), −126.1 (m, 1F), −134.0 (m, 1F). FT-IR (KBr): *ν* (cm^−1^): 2998, 2947, 2845, 1834, 1731, 1499, 1299, 1138, 984, 838, 812, 751.

### Crosslinking of PTFVOPMA-*co*-PMMA

PTFVOPMA-*co*-PMMA copolymer was dissolved in ethyl acetate and the solution was then spin-cast onto the freshly cleaned glass substrate followed by heat treatment at 30 °C for 30 min, 40 °C for 30 min and 65 °C for 1 h. The obtained thin film was placed into a beaker and then the beaker was placed into a tube furnace, followed by evacuating and backfilling with N_2_ three times. The temperature of tube furnace was raised to 100 °C with a rate of 5 °C min^−1^. After keeping at 100 °C for 30 min, the temperature was raised to 150 °C with a rate of 5 °C min^−1^. After keeping at 150 °C for 30 min, the temperature was raised to 180 °C with a rate of 5 °C min^−1^. After reacting at 180 °C for 10 h, the furnace was cooled and crosslinked PTFVOPMA-*co*-PMMA copolymer film was obtained. FT-IR (KBr): *ν* (cm^−1^): 2998, 2950, 1732, 1501, 1488, 1452, 1276, 1199, 1150, 969, 842, 807, 752.

### Water absorption of crosslinked PMMA-based copolymer

The water uptake experiments were carried out as follows: sample films (PMMA and crosslinked PTFVOPMA-*co*-PMMA) were dried *in vacuo* at 55 °C for 24 h and a constant weight (±0.0001 g) can be obtained for the film (about 0.5 g). The films were immersed in ultra-pure water at 25 °C. At certain interval, the films were then taken out of water and the water absorbed on the surface was wiped off by a cleaning cloth. The weight of the films was weighed immediately.

## Results and discussion

### Synthesis of TFVOPMA monomer

TFVOPMA, a methacrylate monomer containing aryl TFVE moiety, was synthesized *via* four steps using 4-methoxylphenol and 1,2-dibromotetrafluoroethane as starting materials. Firstly, hydroxyl of 4-methoxylphenol was transformed into –OCF_2_CF_2_Br group *via* the fluoroalkylation with BrCF_2_CF_2_Br. Subsequent demethylation of resultaning compound by BBr_3_ provided 4-(2-bromo-1,1,2,2-tetrafluoroethoxy)phenol, which was treated with methacryloyl chloride to obtain 4-(2-bromo-1,1,2,2-tetrafluoroethoxy)phenyl methacrylate. Finally, –OCF_2_CF_2_Br moiety of the methacrylate was converted to –OCF=CF_2_ functionality by Zn-mediated elimination to give the target methacrylate monomer, 4-((1,2,2-trifluorovinyl)oxy)phenyl methacrylate (TFVOPMA).

**Fig. 1:**
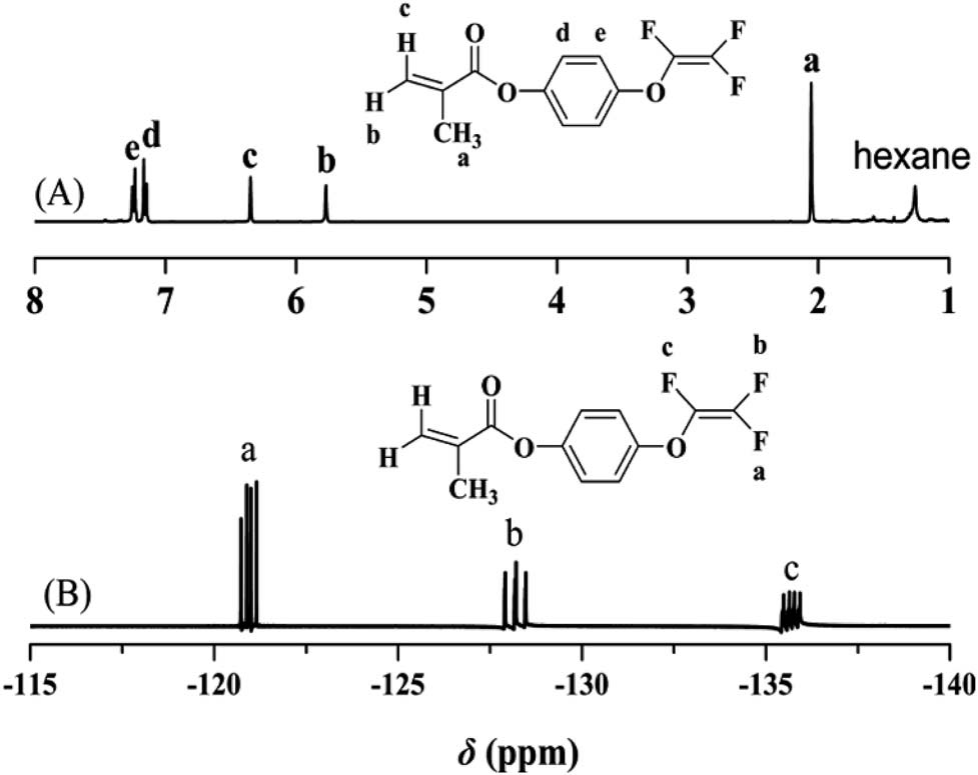
^1^H (A) and ^19^F (B) NMR spectra of TFVOPMA in CDCl_3_.

**Fig. 2:**
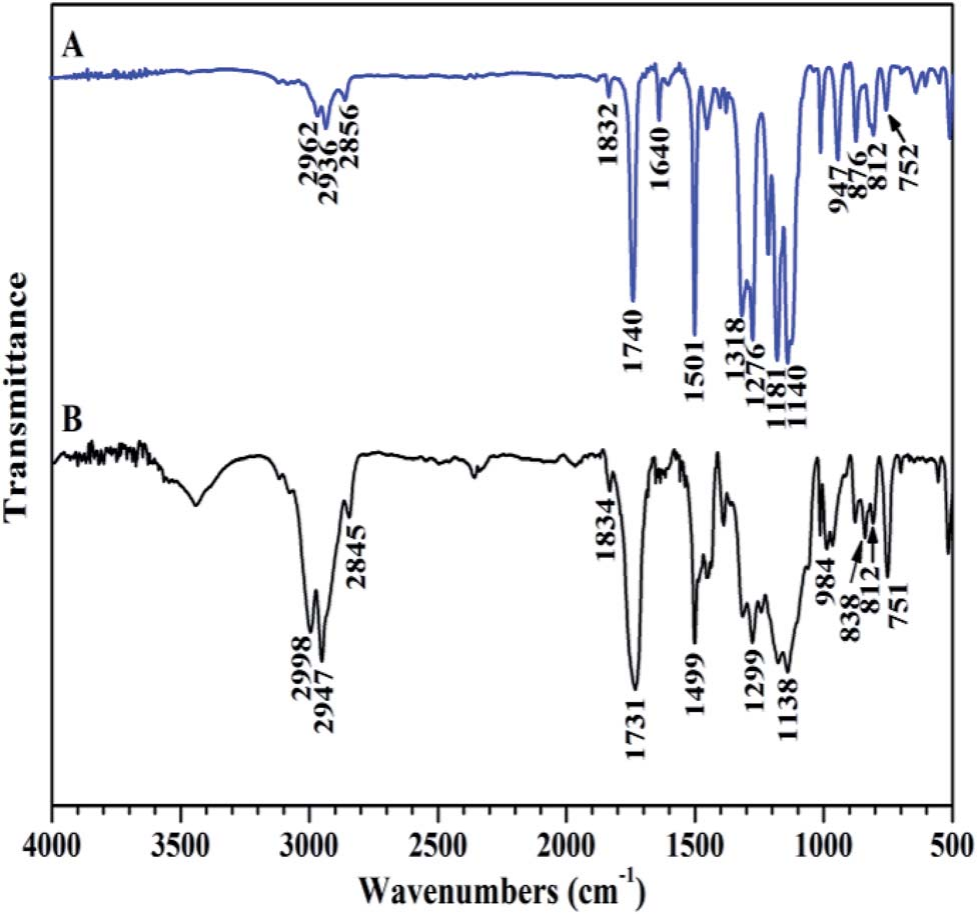
FT-IR spectra of TFVOPMA (A) and PTFVOPMA-*co*-PMMA (B).

The chemical structure of TFVOPMA monomer was characterized by FT-IR, ^1^H NMR and ^19^F NMR. The characteristic peaks of double bond are found to be located at 5.75 (‘b’) and 6.31 (‘c’) ppm in ^1^H NMR spectrum of TFVOPMA (Fig. 1A). The proton resonance signal of methyl linked to the double bond appears at 2.03 ppm (‘a’), while the resonance signals at 7.17 (‘d’) and 7.23 ppm (‘e’) belong to phenyl protons. In ^19^F NMR spectrum of TFVOPMA (Fig. 1B), characteristic peaks of –OCF=CF_2_ group appear at −120.3, −127.8 and −135.3 ppm, respectively. And the appearance of the peak at 1832 cm^−1^ in FT-IR spectrum of TFVOPMA (Fig. 2A) further confirms the presence of –OCF=CF_2_ group. The sharp peak located at 1740 cm^−1^ was attributed to stretching vibration of carbonyl, while the peak at 1640 cm^−1^ belongs to the stretching vibration of double bond. The absorption at 812 cm^−1^ indicates *para*-substitution of benzene ring. All these results confirm the successful synthesis of TFVOPMA, the methacrylate monomer with TFVE moiety.

### Preparation and crosslinking of PTFVOPMA-*co*-PMMA copolymer

Compared to living/controlled polymerization, conventional free radical polymerization allows achieving higher molecular weights and the related industrial technology is mature. Therefore, free radical copolymerization of MMA and TFVOPMA was conducted in 2-butanone at 60 °C using AIBN as initiator to provide the corresponding random copolymer, poly(4-((1,2,2-trifluorovinyl)oxy)phenyl methacrylate)-*co*-poly(methyl methacrylate) (PTFVOPMA-*co*-PMMA).

**Fig. 3:**
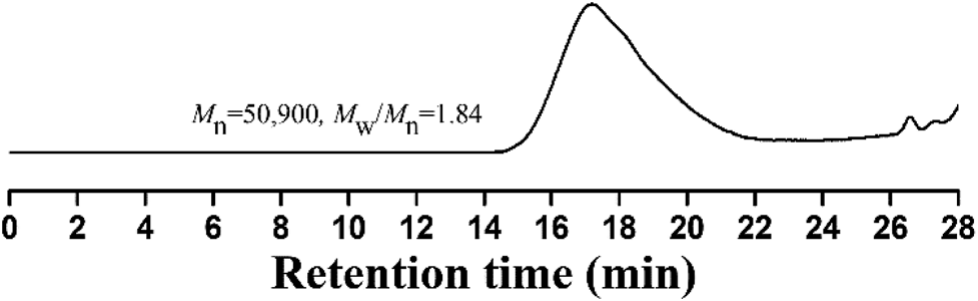
GPC curve of PTFVOPMA-*co*-PMMA in THF.

**Fig. 4:**
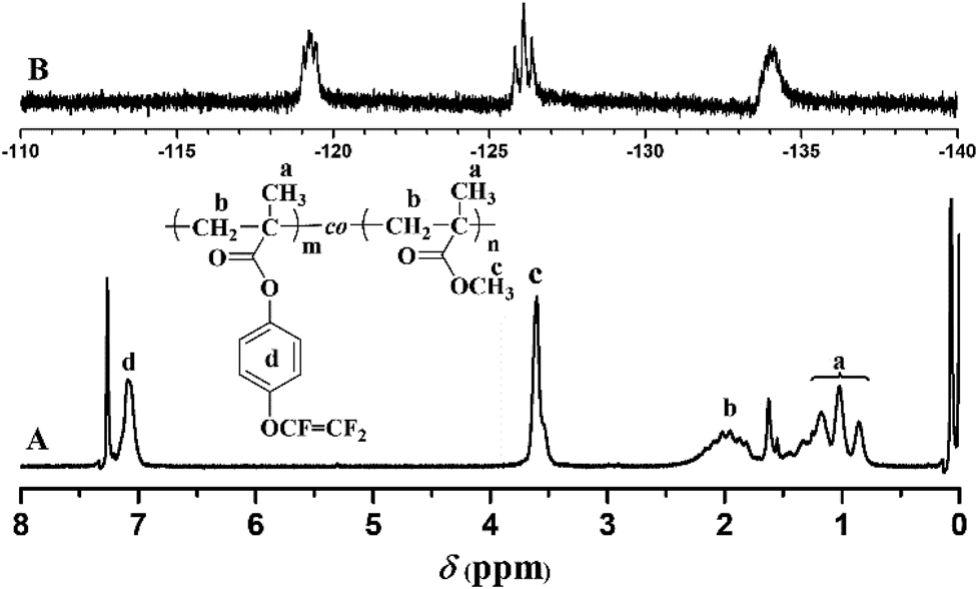
^1^H (A) and ^19^F (B) spectra of PTFVOPMA-*co*-PMMA copolymer in CDCl_3_.

The resulting copolymer was characterized by GPC, FT-IR, ^1^H NMR and ^19^F NMR. GPC retention curve of PTFVOPMA-*co*-PMMA shows a unimodal elution peak with a relatively broad molecular weight distribution of 1.84 and the molecular weight of PTFVOPMA-*co*-PMMA is about 50 900 g mol^−1^ (Fig. 3). In the FT-IR spectrum of PTFVOPMA-*co*-PMMA (Fig. 2B), the typical signal of double bond at 1640 cm^−1^ disappeared after polymerization and the signal of TFVE was located at 1834 cm^−1^, which indicated that TFVE group was not affected during the copolymerization of TFVOPMA and MMA. Other typical signals such as 1731 (C=O, symmetric stretch), 1499 (phenyl ring, stretch), 812 and 838 (Ar–H deformation) are visible and the stretching vibration of –CH_3_ (2998 cm^−1^) and –CH_2_ (2947 and 2845 cm^−1^) become stronger, which also illustrate the preparation of PTFVOPMA-*co*-PMMA copolymer. Fig. 4A shows ^1^H NMR spectrum of PTFVOPMA-*co*-PMMA copolymer, which also showed the disappearance of proton resonance signal of double bond. The peaks of polymethacrylate backbone appeared at 1.85–2.04 ppm (‘b’) and 0.84, 1.02, 1.18 ppm (‘a’), corresponding to the protons of methylene and methyl in –C*H*_2_CC*H*_3_ moiety. The peak at 3.58 ppm (‘c’) is attributed to 3 protons of methoxy group, while the peaks at 7.11 ppm (‘d’) belongs to the protons of phenyl. Therefore, the composition of this copolymer (*N*_TFVOPMA_/*N*_MMA_) was about 1 : 1.93, which was calculated according to the equation (*N*_TFVOPMA_/*N*_MMA_ = 3*S*_d_/4*S*_c_, *S*_c_ and *S*_d_ are the integration area of peak ‘c’ at 3.58 ppm and peak ‘d’ at 7.11 ppm in Fig. 3A). Moreover, the existence of TFVE group appeared at −119.2, −126.1 and −134.0 ppm in ^19^F NMR spectrum (Fig. 4B) further confirmed the preservation of TFVE groups in PTFVOPMA-*co*-PMMA copolymer.

All the above evidence verified that TFVE group did not affect the radical copolymerization of TFVOPMA and MMA and the PMMA-based copolymer containing TFVE aryl ether moieties has been successfully prepared.

### Preparation and properties of crosslinked PMMA-based copolymer

**Fig. 5:**
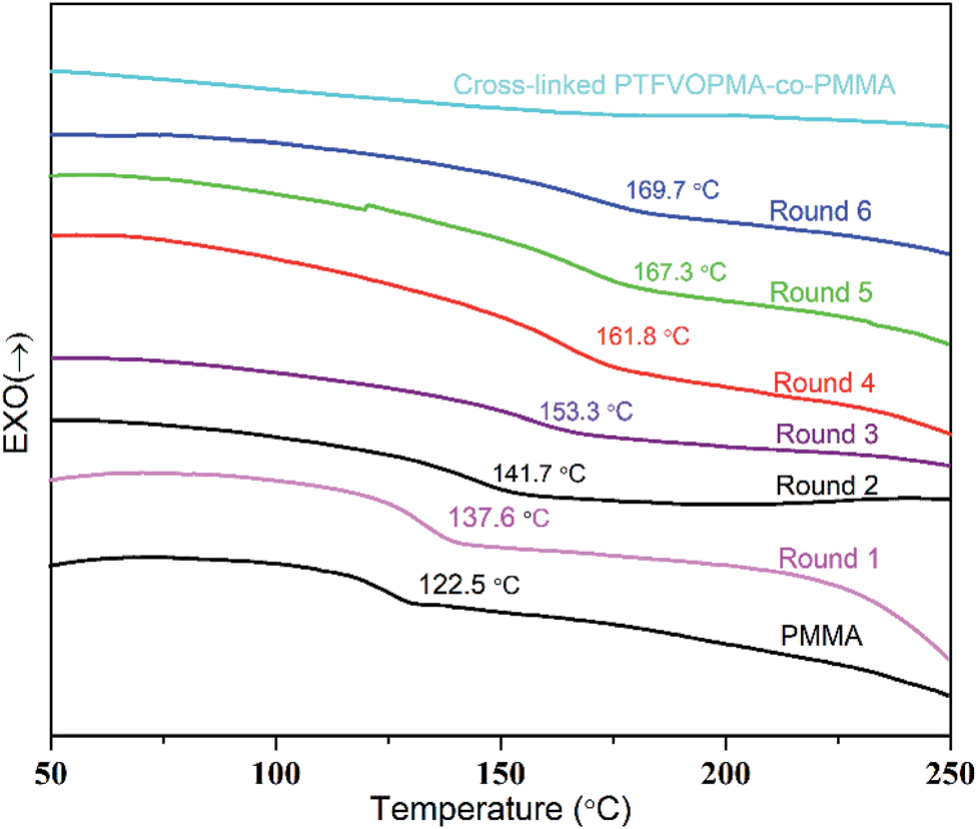
DSC curves (in N_2_) of PMMA, PTFVOPMA-*co*-PMMA and crosslinked PTFVOPMA-*co*-PMMA with a heating rate of 10 °C min^−1^.

Trifluorovinyl ether groups (–OCF=CF_2_) can form perfluorocyclobutane units *via* [2π + 2π] cycloaddition reaction at high temperature, thus we firstly monitored this cross-linking reaction by DSC. As we can see from Fig. 5, *T*_g_ of PTFVOPMA-*co*-PMMA was about 137.6 °C in round 1, higher than that of PMMA (122.5 °C). We supposed that the introduction of rigid benzene ring with large volume makes rotational hindrance of polymeric chain difficult, which leads to higher *T*_g_ of PTFVOPMA-*co*-PMMA. Interestingly, *T*_g_ of PTFVOPMA-*co*-PMMA increased with the scan times and the values of *T*_g_ reached 169.7 °C in the sixth scan. The phenomenon demonstrated that [2π + 2π] cycloaddition reaction of trifluorovinyl ether groups in PTFVOPMA-*co*-PMMA copolymer occured at higher temperature during the DSC measurement and the formed PFCB cross-linking unit further limited the mobility of polymer chains, endowing the polymer with higher *T*_g_.

**Fig. 6:**
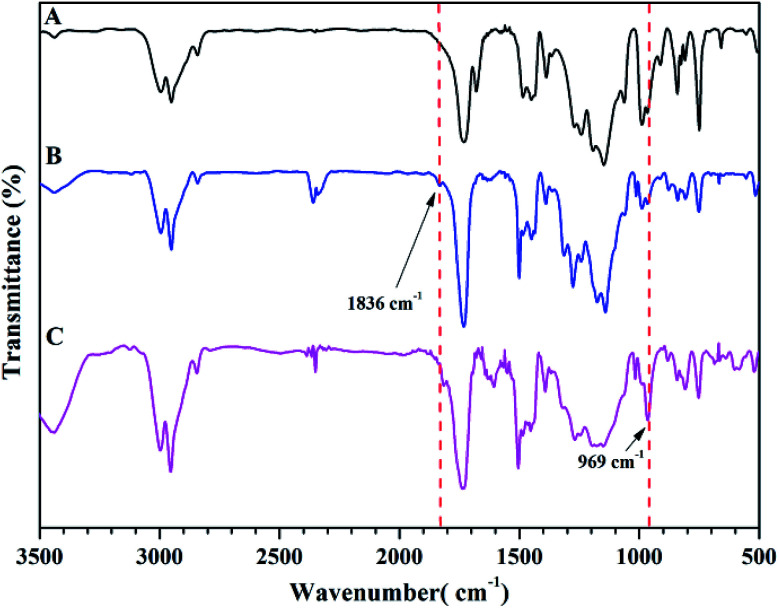
FT-IR spectra of PMMA (A), PTFVOPMA-*co*-PMMA (B) and crosslinked PTFVOPMA-*co*-PMMA (C).

Next, we conducted thermal crosslinking process of PTFVOPMA-*co*-PMMA copolymer. Firstly, thin film of PTFVOPMA-*co*-PMMA was obtained by dissolving PTFVOPMA-*co*-PMMA into ethyl acetate and spin-casting the solution onto the freshly cleaned glass substrate followed by heat treatment at 30 °C for 30 min, 40 °C for 30 min and 65 °C for 1 h. Then, this film was performed in a tube furnace under N_2_ at 180 °C for 10 h, affording a transparent film with a pale yellow color. From FT-IR spectrum of PTFVOPMA-*co*-PMMA after thermal crosslinking (Fig. 6C), the peak at 1836 cm^−1^ corresponding to TFVE group disappeared and a new peak at 969 cm^−1^ corresponding to PFCB ring was found, which indicated the formation of PFCB aryl ether moieties in the copolymer by thermal cycloaddition of aryl TFVE groups. Other typical characteristic peaks can also be seen in FT-IR spectrum after crosslinking (Fig. 6C).

**Fig. 7:**
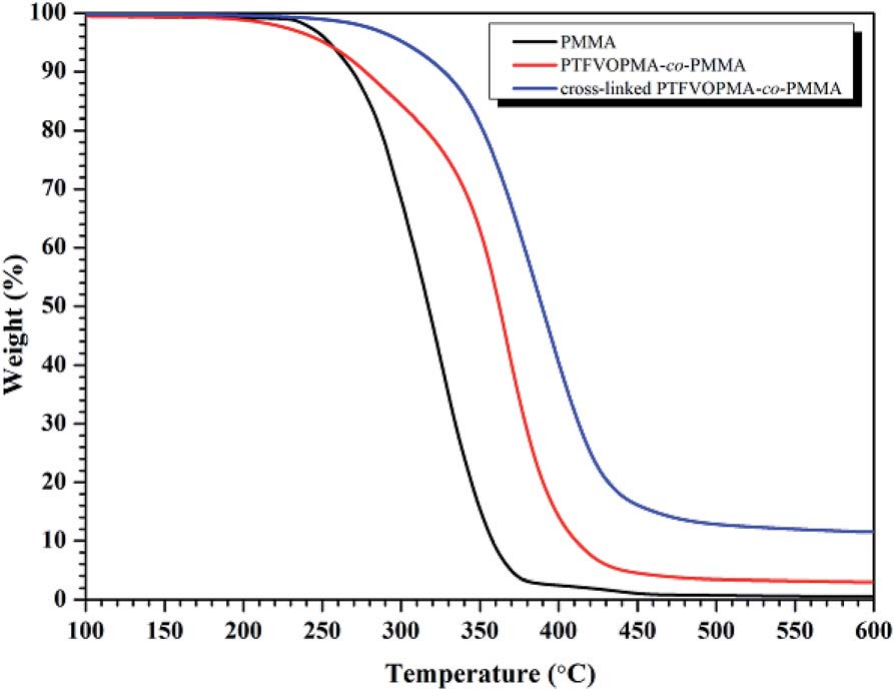
TGA curves (in N_2_) of PMMA, PTFVOPMA-*co*-PMMA and crosslinked PTFVOPMA-*co*-PMMA with a heating rate of 10 °C min^−1^.

As it can be seen from Fig. 5, no *T*_g_ was found on the DSC curve of crosslinked PTFVOPMA-*co*-PMMA below 250 °C, that is, the resultant crosslinked polymer is in glass state below 250 °C, which demonstrated that the formed network with PFCB aryl ether moieties as crosslinking segment largely improved the heat resistance of the obtained PMMA-based copolymer. Thermal degradation of PTFVOPMA-*co*-PMMA before and after crosslinking was investigated by TGA as shown in Fig. 7. The 5% weight loss temperatures (*T*_d,5%_) for PMMA, PTFVOPMA-*co*-PMMA and crosslinked PTFVOPMA-*co*-PMMA are 254 °C, 250 °C and 301 °C, respectively; while the 10% weight loss temperatures (*T*_d,10%_) are 269 °C, 276 °C and 326 °C, respectively. PTFVOPMA-*co*-PMMA containing TFVE groups shows lower thermal degradation temperature at first stage before *T*_d,6%_ (257.6 °C) as compared to that of PMMA, which may be attributed to the polarity and softness of TFVE group. However, PTFVOPMA-*co*-PMMA containing TFVE groups shows higher *T*_d_ at late stage (after *T*_d,6%_ = 257.6 °C), which may be attributed to the formation of enough PFCB rings at higher temperature. As we can see, crosslinked PTFVOPMA-*co*-PMMA with PFCB aryl ether moieties as crosslinked units shows higher thermal stability compared to PMMA and PTFVOPMA-*co*-PMMA. This can be explained that the formation of PFCB ring after thermal treatment decreases the activities of PMMA main chain because of its large volume, and three-dimensional network structure limits the mobility and rotation ability of polymer chain, which may improve the thermal stability of the resultant PMMA-based copolymer.

**Fig. 8:**
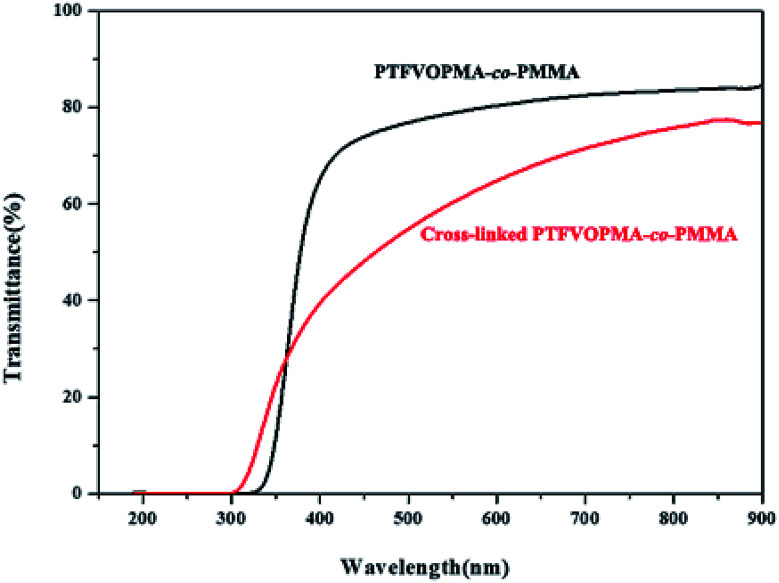
UV/vis spectra of PTFVOPMA-*co*-PMMA and crosslinked PTFVOPMA-*co*-PMMA films.

The light transmittance of PTFVOPMA-*co*-PMMA and cross-linked PTFVOPMA-*co*-PMMA films were investigated by UV/vis spectroscopy. As shown in Fig. 8, the transmittance of PTFVOPMA-*co*-PMMA film are 74% at 500 nm and 80% at 600 nm, and the transmittance of the crosslinked film decreased to 62.5% at 600 nm.

**Fig. 9:**
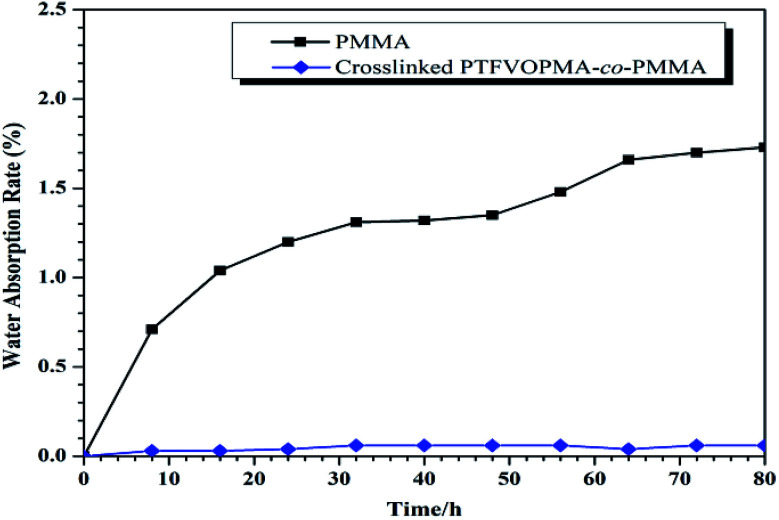
Water absorption rate of PMMA and crosslinked PTFVOPMA-*co*-PMMA films.

Among the unique properties of fluorinated polymers, low water absorption is a typical one. The moisture resistance was evaluated by measuring the weight change of PMMA and crosslinked PTFVOPMA-*co*-PMMA films after immersing into pure water for a certain time (Fig. 9). It is noted that the water absorption of crosslinked PTFVOPMA-*co*-PMMA film greatly decreased in comparison with pure PMMA (0.03% *vs.* 1.73% after 8 h). Furthermore, the water absorption of crosslinked PTFVOPMA-*co*-PMMA film retained below 0.06%, while that of PMMA increased with the immersing time. The decreasing water absorption could be due to the highly hydrophobic nature of aryl PFCB moieties.

## Conclusions

In summary, a methacrylate monomer containing aryl TFVE group, TFVOPMA, was synthesized and copolymerized with MMA to provide the copolymer containing aryl TFVE groups, PTFVOPMA-*co*-PMMA, which was treated at 180 °C to give crosslinked network PTFVOPMA-*co*-PMMA copolymer *via* the formation of PFCB moieties. The glass transition temperature of crosslinked network PTFVOPMA-*co*-PMMA copolymer is high and they are thermally stable with less water absorption.

## Conflicts of interest

There are no conflicts to declare.

## Supplementary Material
